# Local Application of Chloroform in Epididymitis

**Published:** 1890-05

**Authors:** 


LOCAL APPLICATION OF CHLOROFORM IN EPIDIDYMITIS.—Dr. Theodore Clemens, of Frankfort, in an interesting paper communicated to the *Algemeine Medicinische Central Zeitung*, describes the great benefit he has obtained in cases of epididymitis, both specific and non-specific, by means of chloroform locally applied. He regards as most unsatisfactory the treatment of the affection by other methods as compared with his own, which he has employed now for a great many years. It consists in laying some cotton wool saturated with chloroform and spirit at the bottom of a large glass vessel, into which the genitals are then put and packed round with dry cotton wool, the buttocks and thighs forming a cover, this application being continued for from fifteen to twenty-five minutes, and repeated two or three times a day. Pathologically, he considers venous congestion of the epididymis and the cord through retention of the semen a predisposing cause of the disease. He also considers epididymitis as very likely to occur when gonorrhœa has been contracted in excessive venery. He mentions a case of treatment by chloroform, thirty-six years ago, not of epididymitis, but of periodical "heat" occurring in the human subject. The man used to suffer periodically from a form of orchitis, during which the testes felt hot and swollen, and the plexus pampiniformis was full of turgescent like a varicocele. He was ordered the local application of chloroform three times a day, from fifteen to twenty-five minutes each time, but the first time he bore the chloroform for nearly thirty-five minutes, after which the pain of the severe attack completely ceased and the swelling considerably decreased. This treatment lasted three days, during which time he was able to walk about, the cotton wool which had been used for the chloroform being put into the suspensory bandage and the testes covered with it. After that both the swelling and sensibility disappeared. Another case is mentioned, where epididymitis had been caused by the continuous pressure of a rudder handle on the hypogastrium, in which similar treatment proved entirely successful. Again, a class of cases that is usually very difficult to treat, viz.: that of gonorrhœal orchitis, seems to have proved fairly tractable when managed with the help of chloroform. Here one of the first signs of improvement was frequently the re-establishment of an old discharge, which was soon cured simultaneously with epididymitis.—*Lancet.*—*Gaillard's Medical Journal.*

